# Post-caesarean Section Headache: A Case Report of Post-dural Puncture Headache and Cerebral Venous Thrombosis Following Epidural Anaesthesia

**DOI:** 10.7759/cureus.60183

**Published:** 2024-05-13

**Authors:** Rachael S Lim, Ethan K Chan, Partha P Das, Tunde Ibrahim

**Affiliations:** 1 Critical Care, Goulburn Valley Health, Shepparton, AUS; 2 General Medicine, Goulburn Valley Health, Shepparton, AUS

**Keywords:** epidural anaesthesia, dural venous sinus thrombosis, cerebral venous thrombosis, autologous blood patch, post dural puncture headache, post-partum headache

## Abstract

Post-dural puncture headache (PDPH) is a common complication of epidural and spinal anaesthesia in obstetric medicine. In rare cases, PDPH can be associated with complications such as cerebral venous thrombosis (CVT) as well. We discuss a recent case of a young female who developed PDPH and CVT concurrently after undergoing epidural anaesthesia for initially uncomplicated labour and delivered via an emergency caesarean section. She developed an orthostatic headache a few hours post administration of the epidural anaesthetic, which was initially treated as a suspected PDPH by giving simple analgesia and caffeine. Her symptoms did not improve and she underwent further neuroimaging, which revealed the development of a CVT. Despite the prompt administration of enoxaparin, the headache persisted and did not respond to increased doses of analgesia. After deliberation and inter-departmental discussion, an epidural blood patch was performed, leading to the prompt resolution of the headache.

This report highlights a rare concurrence of PDPH and CVT, causing a diagnostic dilemma that resulted in treatment delays for the patient. Treating both conditions raises difficult practical questions, especially regarding the use of an epidural blood patch as opposed to anticoagulation. Given the risk of fatal complications such as venous cerebral infarction, seizures, and subdural hematoma, prompt treatment of both PDPH and CVT is strongly recommended. The multifactorial mechanism by which CVT develops with intracranial hypotension and PDPH also makes it essential for clinicians to keep an open mind when managing post-caesarean headaches, requiring inter-departmental cooperation to ensure optimal patient outcomes.

## Introduction

Post-dural puncture headache (PDPH) following spinal and epidural anaesthesia is a rare entity, with a typical incidence of 3%, but it affects up to 9% of patients when certain sub-optimal factors are present. These factors include needle type and size, age, gender, pregnancy, previous post-spinal headache history, number of procedural attempts, midline versus lateral approach, type of local anaesthetic solution, and operator proficiency [1-5]. PDPH is most prevalent in obstetric patients, with an incidence rate of about 81-88% following unintentional dural puncture (UDP) with an epidural needle for labour analgesia, despite the low risk (1-6%) of UDP in general [6-9].

Aside from headaches, intracranial hypotension may result in other complications such as cerebral venous thrombosis (CVT) due to changes in intracranial structure and positioning, blood and cerebral spinal fluid (CSF) flow dynamics, and caudal brain movement [10-12]. This case report serves as a reminder for clinicians to consider other causes of post-caesarean headache, with CVT as a possible differential diagnosis. Of particular note was the lack of obvious dural puncture on administration of the epidural anaesthesia, even though in hindsight, the clinical signs of the case could have indicated PDPH as the most likely cause.

## Case presentation

A 23-year-old previously healthy primigravida woman underwent epidural anaesthesia for an initially uncomplicated labour. The epidural insertion was routine and uneventful, with no obvious dural puncture noted by the anaesthetist. A test bolus of 5 ml ropivacaine 0.2% + fentanyl 2 mcg/ml was administered, followed by an additional 5 ml of the same drugs as the initial dose after the anaesthetist confirmed the epidural to be effective. Throughout the course of labour, a total of 19 ml of ropivacaine 0.2% + fentanyl 2 mcg/ml was administered via the patient-controlled epidural analgesia.

The patient's labour progressed to an emergency caesarean section due to obstructed labour and abnormal cardiotocography (CTG). Top-up of the epidural analgesia with lignocaine 2% with adrenaline and fentanyl 100 mcg was administered as adequate analgesia for the procedure. There were no complications from the caesarean section, with an estimated blood loss of only 400 mL. A healthy baby girl was delivered and both the baby and the mother were transferred back to the maternity ward for continual care. Five hours after returning to the ward (12 hours after the epidural catheter insertion), the patient developed a persistent, incapacitating orthostatic headache, significantly impairing her ability to care for herself and her newborn child. This was unusual, as the patient had neither any significant prior headache history nor any risk factors in her background or family history. 

Upon examination at the start of the headache, the patient's vital signs were stable, and no neurological abnormalities suggestive of meningitis were noted. The patient was afebrile at that time, but it was noted that she had spiked temperatures, ranging from 37.9 °C to 38.2 °C, twice after returning from theatre. A distinct positional aspect related to the headache was also noted, with the headache worsening on sitting up and improving on lying supine. Initial investigations revealed mildly elevated inflammatory markers (Table 1) but were otherwise unremarkable.

**Table 1:** Pathology results of the patient

Variables	Day 3 (since admission)	Day 4	Day 5	Day 9	Day 13	Reference range
Haemoglobin	97	96	99	108	103	115 - 155 g/L
White cell count	13.1	11.6	10	10.2	10.2	4.0 - 12.0 x 10^9^/L
Platelet count	265	292	339	458	430	150 - 400 x 10^9^/L
Neutrophils	9.6	7.8	7.2	6.8	6	2.0 - 8.0 x 10^9^/L
C-reactive protein	195	135	93.6	30.3	31.8	<5.0 mg/L

The treatment for suspected PDPH and possible postoperative sepsis was initiated by the obstetrician, including simple analgesia (i.e., paracetamol and NSAIDs), intravenous fluids, and antibiotics. Adjustments in management were also made by the Acute Pain team, including analgesia titration and caffeine administration. However, the patient's symptoms persisted. Given the refractory nature of her symptoms, the patient was referred to the medical team for further evaluation. Their initial clinical assessment supported a suspected PDPH secondary to UDP and CSF leak. Upon extensive discussion with the anaesthetic team and differing diagnostic opinions, the decision was made to conduct further workup to aid the diagnosis.

**Figure 1:**
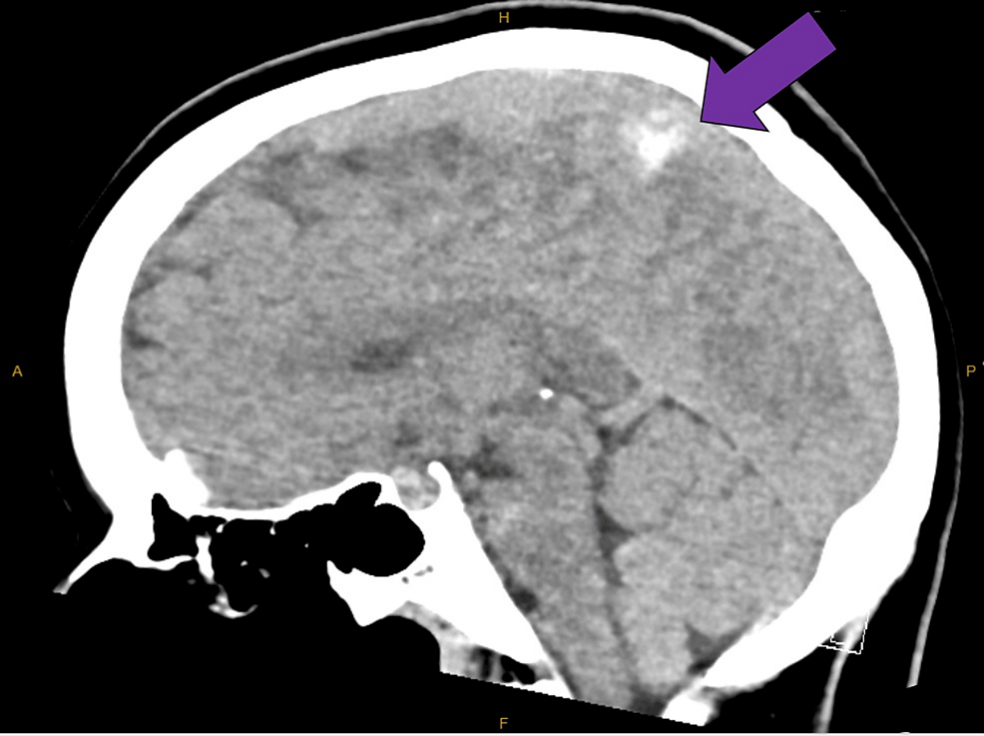
Non-contrast CT brain showing blood clot in the superior sagittal venous sinus (purple arrow) CT: computed tomography

**Figure 2:**
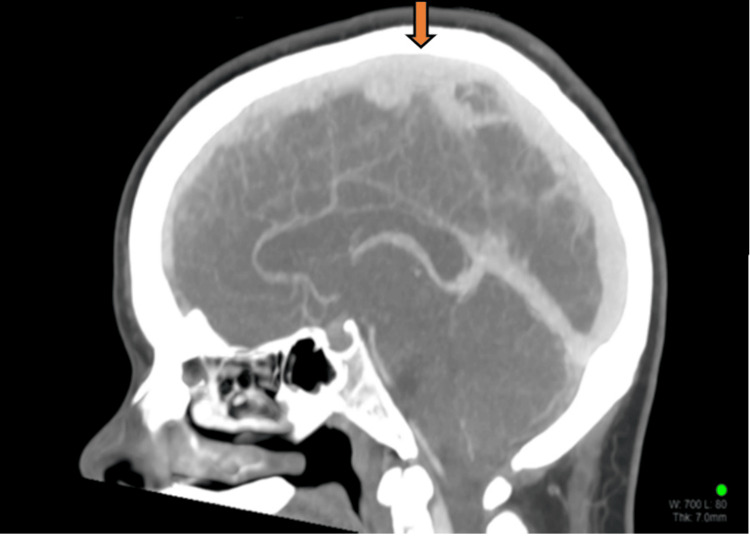
CT cerebral venogram showing the filling defect in the superior sagittal venous sinus due to a thrombus (orange arrow) CT: computed tomography

**Figure 3:**
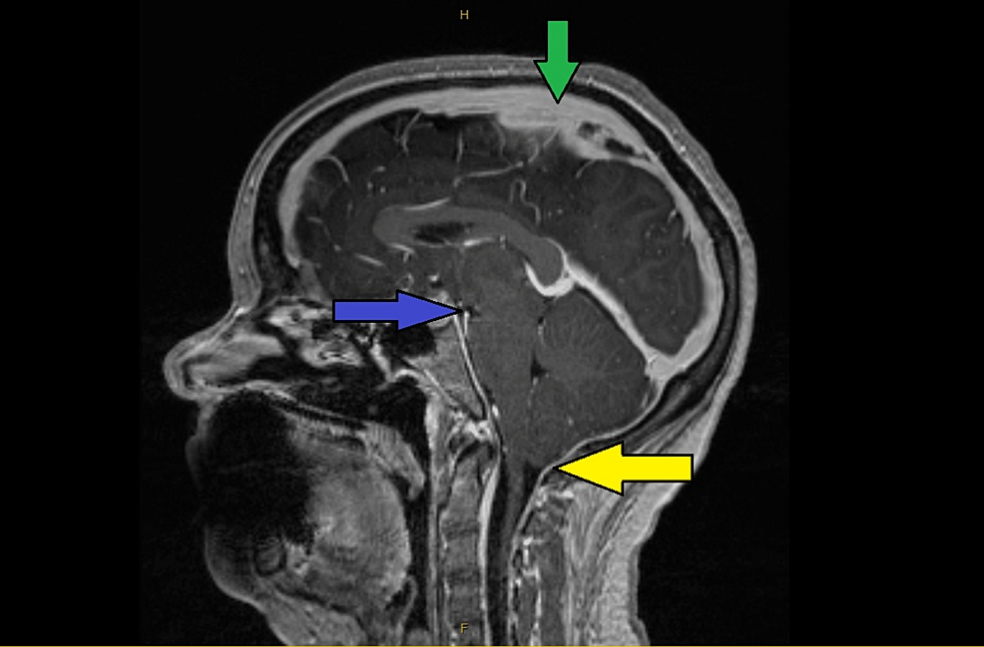
MRI venogram of the brain The image showed features of intracranial hypotension and superior sagittal venous thrombus. The green arrow points to the filling defect in the superior sagittal venous sinus due to a thrombus. The blue arrow shows reduced mamillopontine distance and reduced pontomesencephalic angle. The yellow arrow points to mild tonsillar herniation due to caudal cerebral tonsil descent MRI: magnetic resonance imaging

Imaging studies, notably a CT brain scan, revealed a superior sagittal venous thrombus (Figure 1). This was confirmed with a CT-venography (Figure 2). The working diagnosis for the headache thus changed to being a CVT and therapeutic enoxaparin was commenced. At this point, an external neurosurgical unit was consulted to seek management advice for the patient. However, even after five days of this treatment, the patient continued to experience the same debilitating orthostatic headaches. An MRI venography was then performed to further characterize the CVT, which unexpectedly also revealed signs of intracranial hypotension with mild tonsillar herniation (Figure 3). This led to further contradictory opinions on diagnosis among the various departments involved.

As part of a collaborative effort, an epidural blood patch was performed by the Anaesthetic team 10 days after the patient's initial obstetric admission; 25 mL of autologous blood was injected into the L3/L4 intervertebral space, resulting in a near-immediate improvement of symptoms. The patient was observed to be able to mobilize and care for her newborn child, reporting complete resolution of her headache 12 hours after the epidural blood patch administration.

Following symptom resolution, the patient was cleared for discharge home with her newborn child, with appropriate follow-up arranged to treat and monitor her CVT.

## Discussion

We reported a PDPH case associated with CVT in a patient post-caesarean section due to UDP. The patient started experiencing the headache a few hours post-procedure and it was orthostatic in nature, similar to presentations of PDPH in other studies. About 90% of PDPH cases occur within 72 hours of the procedure, but some studies have described cases where symptoms occurred up to two weeks post-procedure. Based on our analysis of other cases in the literature, the severity of the headache is higher if symptoms begin within 24 hours. Resting in a supine position and refraining from standing or raising the head from the bed can help improve these symptoms [13,14].

The etiopathogenesis of PDPH is unclear but likely related to intracranial hypotension due to the leakage of CSF through the dural hole created by the needle. Some of the intracranial changes that may contribute to PDPH are observable in neuroimaging. These include low CSF pressure due to loss outpacing the production of CSF, the descent of the brain in the upright position leading to compensatory meningeal venodilation, blood volume expansion, sagging of intracranial structures, and stretching of sensory intracranial nerves [15-20]. These intracranial changes may also contribute to the development of other complications such as CVT, subdural hematoma, and CSF accumulation, all of which have also been associated with PDPH.

Similar to our case, Garcia-Carreira et al. [20] have reported two cases of CVT complicating PDPH. Haritanti et al. [19] also noted that spontaneous intracranial hypotension (SIH) is a risk factor for CVT, seen in ~2% of patients. As in our case, the presence or development of CVT in PDPH may be associated with little or no change in the characteristics of the orthostatic headache. The mechanisms behind SIH leading to CVT include those described by the Monro-Kellie Doctrine [21], which states that in a closed compartment such as the intracranial and dural space, any loss of one component will be compensated for by an increase in at least another one. Therefore, the lost CSF volume will be replaced by an increase in the most easily expansible component, which is venous blood. The venous engorgement leads to the appearance of a thickened dura and the dilation of cerebral veins and sinuses, resulting in a decrease in blood flow velocity. Studies using transcranial Doppler ultrasound have shown that blood flow velocities in the sagittal venous sinus decrease by about 47% post-lumbar puncture (LP) [22,23].

SIH is also associated with rostrocaudal descent of the brain due to the loss of CSF buoyancy. This results in a negative intracranial pressure gradient, which can cause damage to the venous endothelium by stretching the cerebral vessels [19]. Additionally, the loss of CSF reduces its absorption into the cerebral venous sinuses, resulting in increased blood viscosity in the venous compartment. All of these changes likely contribute to the formation of CVT, especially in patients with risk factors for thrombosis, including hereditary thrombophilia, caesarean delivery, pregnancy-related hypertension, and combined oral contraceptive use [24].

The clinical diagnosis of PDPH involves identifying the typical orthostatic headache occurring within 72 hours after a dural puncture procedure. Amorim et al. [25] have reported a latency time of 6-72 hours between LP and the onset of headache, with most headaches lasting 5-15 days. If symptoms are atypical, not responding to the recommended PDPH treatments, or lasting longer than the average seven days, other causes need to be excluded. As our patient failed to respond to the usual management for 48 hours, other possible causes of her headache were duly considered and investigated. Investigations should be tailored to differential diagnoses such as CVT, pregnancy-induced hypertension, posterior reversible encephalopathy syndrome (PRES), meningitis, migraine, intracranial haemorrhages, and brain tumours [10-13].

The gold standard diagnostic investigation in PDPH and CVT is neuroimaging, with CT or MRI scans being the most commonly used modalities. Other investigations like radio-nuclear scans are sometimes used to study the CSF flow dynamics. The findings suggestive of PDPH include small ventricles, rostrocaudal displacement (sagging) of the brain, engorged cerebral venous sinuses, subdural CSF collections, pituitary enlargement, and diffuse meningeal enhancement [26-29]. Neuroimaging can also reveal certain complications of PDPH, such as CVT or subdural haematoma. If meningitis is strongly suspected, diagnostic LP should be avoided as this can worsen any existing PDPH. If an LP is performed, a low CSF opening pressure or dry tap is indicative of intracranial hypotension. However, the presence of increased CSF protein and lymphocyte count may lead to the condition being misdiagnosed as aseptic meningitis [28].

The treatment of PDPH depends on the severity of the headache and its impact on the patient's ability to function, especially in obstetric cases where caring for a newborn is of utmost concern. For mild cases, conservative treatment with oral analgesia, antiemetics, caffeine, and oral or intravenous hydration are the mainstays of treatment [29]. In debilitating cases (characterized by severe headaches, inability to tolerate sitting upright or negative impact on activities of daily living), patients should be offered an epidural blood patch, as in our patient. In cases with associated CVT, treatment with anticoagulation is recommended [30].

The concurrence of PDPH and CVT raises difficult practical questions about the treatment of the two conditions. On the one hand, the definitive management for severe cases of PDPH is an epidural blood patch. However, the use of anticoagulants in treating CVT can increase the risk of epidural blood patch complications (e.g., subdural haematoma). As such, the risks and benefits need to be carefully assessed. In rare cases of severe CVT, neurosurgical intervention may be indicated [30]. Fortunately, after consultation with neurosurgery, this was not required in our patient.

## Conclusions

This report underscores the complexity of managing post-epidural anaesthesia-related complications, highlighting the significance of promptly recognizing and addressing the rare but serious conditions of PDPH and CVT. The initial treatment in our case targeted PDPH and suspected postoperative sepsis, but further investigation revealed unexpected findings of CVT, requiring ongoing adaptation to new working diagnoses. This case also highlights the importance of a collaborative approach and the need for open-mindedness in clinical practice, particularly when multiple overlying conditions are involved. Multidisciplinary collaboration (particularly between the Obstetric, Anaesthetic, and General Medicine teams) played a pivotal role in identifying and managing these complications, ultimately leading to positive patient outcomes with eventual symptom resolution and discharge home.

## References

[1] DelPizzo K, Cheng J, Dong N (2017). Post-dural puncture headache is uncommon in young ambulatory surgery patients. HSS J.

[2] Choi PT, Galinski SE, Takeuchi L, Lucas S, Tamayo C, Jadad AR (2003). PDPH is a common complication of neuraxial blockade in parturients: a meta-analysis of obstetrical studies. Can J Anaesth.

[3] Vallejo MC, Mandell GL, Sabo DP, Ramanathan S (2000). Postdural puncture headache: a randomized comparison of five spinal needles in obstetric patients. Anesth Analg.

[4] Nath S, Koziarz A, Badhiwala JH (2018). Atraumatic versus conventional lumbar puncture needles: a systematic review and meta-analysis. Lancet.

[5] Naulty JS, Hertwig L, Hunt CO, Datta S, Ostheimer GW, Weiss JB (1990). Influence of local anesthetic solution on postdural puncture headache. Anesthesiology.

[6] Banks S, Paech M, Gurrin L (2001). An audit of epidural blood patch after accidental dural puncture with a Tuohy needle in obstetric patients. Int J Obstet Anesth.

[7] Sprigge JS, Harper SJ (2008). Accidental dural puncture and post dural puncture headache in obstetric anaesthesia: presentation and management: a 23-year survey in a district general hospital. Anaesthesia.

[8] Costa AC, Satalich JR, Al-Bizri E (2019). A ten-year retrospective study of post-dural puncture headache in 32,655 obstetric patients. Can J Anaesth.

[9] Ivanidze J, Zimmerman RD, Sanelli PC (2010). Spontaneous intracranial hypotension followed by dural sinus thrombosis: a case report. Clin Neurol Neurosurg.

[10] Mao YT, Dong Q, Fu JH (2011). Delayed subdural hematoma and cerebral venous thrombosis in a patient with spontaneous intracranial hypotension. Neurol Sci.

[11] Berroir S, Grabli D, Héran F, Bakouche P, Bousser MG (2004). Cerebral sinus venous thrombosis in two patients with spontaneous intracranial hypotension. Cerebrovasc Dis.

[12] Vilming ST, Kloster R (1997). Post-lumbar puncture headache: clinical features and suggestions for diagnostic criteria. Cephalalgia.

[13] Kuntz KM, Kokmen E, Stevens JC, Miller P, Offord KP, Ho MM (1992). Post-lumbar puncture headaches: experience in 501 consecutive procedures. Neurology.

[14] Hannerz J, Ericson K, Bro Skejø HP (1999). MR imaging with gadolinium in patients with and without post-lumbar puncture headache. Acta Radiol.

[15] Bakshi R, Mechtler LL, Kamran S, Gosy E, Bates VE, Kinkel PR, Kinkel WR (1999). MRI findings in lumbar puncture headache syndrome: abnormal dural-meningeal and dural venous sinus enhancement. Clin Imaging.

[16] Ljubisavljevic S, Trajkovic JZ, Ignjatovic A, Stojanov A (2020). Parameters related to lumbar puncture do not affect occurrence of postdural puncture headache but might influence its clinical phenotype. World Neurosurg.

[17] Levine DN, Rapalino O (2001). The pathophysiology of lumbar puncture headache. J Neurol Sci.

[18] Garcia-Carreira MC, Vergé DC, Branera J, Zauner M, Herrero JE, Tió E, Perpinyà GR (2014). Cerebral venous thrombosis in two patients with spontaneous intracranial hypotension. Case Rep Neurol Med.

[19] Mokri B (2001). The Monro-Kellie hypothesis: applications in CSF volume depletion. Neurology.

[20] Canhão P, Batista P, Falcão F (2005). Lumbar puncture and dural sinus thrombosis--a causal or casual association?. Cerebrovasc Dis.

[21] Yoon KW, Cho MK, Kim YJ, Lee SK (2011). Sinus thrombosis in a patient with intracranial hypotension: a suggested hypothesis of venous stasis. a case report. Interv Neuroradiol.

[22] Ferrante T, Latte L, Abrignani G, Russo M, Manzoni GC, Torelli P (2012). Cough headache secondary to spontaneous intracranial hypotension complicated by cerebral venous thrombosis. Neurol Sci.

[23] Amorim JA, Gomes de Barros MV, Valença MM (2012). Post-dural (post-lumbar) puncture headache: risk factors and clinical features. Cephalalgia.

[24] Settipani N, Piccoli T, La Bella V, Piccoli F (2004). Cerebral venous sinus expansion in post-lumbar puncture headache. Funct Neurol.

[25] Kayacan N, Arici G, Karsli B, Erman M (2004). Acute subdural haematoma after accidental dural puncture during epidural anaesthesia. Int J Obstet Anesth.

[26] Wiesemann E, Berding G, Goetz F, Windhagen A (2006). Spontaneous intracranial hypotension: correlation of imaging findings with clinical features. Eur Neurol.

[27] Ahmed SV, Jayawarna C, Jude E (2006). Post lumbar puncture headache: diagnosis and management. Postgrad Med J.

[28] Basurto Ona X, Osorio D, Bonfill Cosp X (2015). Drug therapy for treating post-dural puncture headache. Cochrane Database Syst Rev.

[29] Halker RB, Demaerschalk BM, Wellik KE, Wingerchuk DM, Rubin DI, Crum BA, Dodick DW (2007). Caffeine for the prevention and treatment of postdural puncture headache: debunking the myth. Neurologist.

[30] Kokki M, Sjövall S, Kokki H (2012). Epidural blood patches are effective for postdural puncture headache in pediatrics--a 10-year experience. Paediatr Anaesth.

